# A physiological increase in maternal cortisol alters uteroplacental metabolism in the pregnant ewe

**DOI:** 10.1113/JP272301

**Published:** 2016-07-06

**Authors:** O. R. Vaughan, K. L. Davies, J. W. Ward, M. J. de Blasio, A. L. Fowden

**Affiliations:** ^1^ Centre for Trophoblast ResearchDepartment of Physiology, Development and NeuroscienceUniversity of CambridgeCambridgeCB2 3EGUK

## Abstract

**Key points:**

- Fetal nutrient supply is dependent, in part, upon the transport capacity and metabolism of the placenta.
- The stress hormone, cortisol, alters metabolism in the adult and fetus but it is not known whether cortisol in the pregnant mother affects metabolism of the placenta.
- In this study, when cortisol concentrations were raised in pregnant sheep by infusion, proportionately more of the glucose taken up by the uterus was consumed by the uteroplacental tissues while less was transferred to the fetus, despite an increased placental glucose transport capacity. Concomitantly, the uteroplacental tissues produced lactate at a greater rate.
- The results show that maternal cortisol concentrations regulate uteroplacental glycolytic metabolism, producing lactate for use *in utero*.
- Prolonged increases in placental lactate production induced by cortisol overexposure may contribute to the adverse effects of maternal stress on fetal wellbeing.

**Abstract:**

Fetal nutrition is determined by maternal availability, placental transport and uteroplacental metabolism of carbohydrates. Cortisol affects maternal and fetal metabolism, but whether maternal cortisol concentrations within the physiological range regulate uteroplacental carbohydrate metabolism remains unknown. This study determined the effect of maternal cortisol infusion (1.2 mg kg^−1^ day^−1^ i.v. for 5 days, *n* = 20) on fetal glucose, lactate and oxygen supplies in pregnant ewes on day ∼130 of pregnancy (term = 145 days). Compared to saline infusion (*n* = 21), cortisol infusion increased maternal, but not fetal, plasma cortisol (*P* < 0.05). Cortisol infusion also raised maternal insulin, glucose and lactate concentrations, and blood pH, $P_{CO_{2}}$ and HCO_3_^−^ concentration. Although total uterine glucose uptake determined by Fick's principle was unaffected, a greater proportion was consumed by the uteroplacental tissues, so net fetal glucose uptake was 29% lower in cortisol‐infused than control ewes (*P* < 0.05). Concomitantly, uteroplacental lactate production was > 2‐fold greater in cortisol‐ than saline‐treated ewes (*P* < 0.05), although uteroplacental O_2_ consumption was unaffected by maternal treatment. Materno‐fetal clearance of non‐metabolizable [^3^H]methyl‐d‐glucose and placental *SLC2A8* (glucose transporter 8) gene expression were also greater with cortisol treatment. Fetal plasma glucose, lactate or α‐amino nitrogen concentrations were unaffected by treatment although fetal plasma fructose and hepatic lactate dehydrogenase activity were greater in cortisol‐ than saline‐treated ewes (*P* < 0.05). Fetal plasma insulin levels and body weight were also unaffected by maternal treatment. During stress, cortisol‐dependent regulation of uteroplacental glycolysis may allow increased maternal control over fetal nutrition and metabolism. However, when maternal cortisol concentrations are raised chronically, prolonged elevation of uteroplacental lactate production may compromise fetal wellbeing.

## Key points

- Fetal nutrient supply is dependent, in part, upon the transport capacity and metabolism of the placenta.
- The stress hormone, cortisol, alters metabolism in the adult and fetus but it is not known whether cortisol in the pregnant mother affects metabolism of the placenta.
- In this study, when cortisol concentrations were raised in pregnant sheep by infusion, proportionately more of the glucose taken up by the uterus was consumed by the uteroplacental tissues while less was transferred to the fetus, despite an increased placental glucose transport capacity. Concomitantly, the uteroplacental tissues produced lactate at a greater rate.
- The results show that maternal cortisol concentrations regulate uteroplacental glycolytic metabolism, producing lactate for use *in utero*.
- Prolonged increases in placental lactate production induced by cortisol overexposure may contribute to the adverse effects of maternal stress on fetal wellbeing.

**Abbreviations**
GLUT: glucose transporter
FBPase: fructose‐1,6‐bisphosphatase
G6Pase: glucose‐6‐phosphatase
[^3^H]MeDG: [^3^H]methyl‐d‐glucose
LDH: lactate dehydrogenase

## Introduction

The fetus requires nutrients such as glucose and lactate for both oxidative metabolism and tissue accretion *in utero* (Barcroft *et al*. 1939; Battaglia & Meschia, 1988; Fowden, 1997). These nutrients must ultimately be acquired from the mother, via the placenta. Uterine uptake and transplacental transport of glucose occur by facilitated diffusion (Sibley *et al*. 1997). This depends on the materno‐fetal glucose concentration gradient, the surface area of the placenta for transport and the abundance of facilitative glucose transporters (GLUTs) 1, 3 and 8 in the ovine placenta (Limesand *et al*. 2004; Wooding *et al*. 2005). The net amount of glucose reaching the fetus also depends on the rate of glucose consumption by the uteroplacental tissues for their own metabolic activities (Hay *et al*. 1990). In sheep and other species, about half of the glucose taken up by the gravid uterus is used by the uteroplacental tissues for both oxidative and non‐oxidative metabolism, including glycolysis (Aldoretta & Hay, 1999; Fowden *et al*. 2001). The lactate produced glycolytically is then released into both maternal and fetal circulations (Sparks *et al*. 1982). The energy produced by the uteroplacental tissues is used, in part, for the active transport and transamination of amino acids needed to meet the fetal nutrient requirements (Battaglia & Regnault, 2001). Thus, uteroplacental metabolism is an important determinant of the fetal nutrient supply.

Glucocorticoids, like cortisol, regulate metabolism in adult and fetal tissues (McMahon *et al*. 1988; Fowden *et al*. 1998). Glucocorticoid concentrations rise normally in late pregnancy (Bell *et al*. 1991; Fowden *et al*. 1998; Jensen *et al*. 2002 *a*) but may be elevated before term if the mother is exposed to stresses such as malnutrition, infection or transport (Fowden & Silver, 1985; Edwards & McMillen, 2001; Kabaroff *et al*. 2006; Miranda‐de la Lama *et al*. 2012). When maternal cortisol concentrations are increased exogenously in pregnant sheep for ≥ 10 days during late gestation, there are alterations in maternal glucose concentrations and uterine blood flow, which are associated with impaired fetoplacental growth and poor fetal viability (Jensen *et al*. 2005, 2010; Keller‐Wood *et al*. 2014). In part, these changes may relate to alterations in uteroplacental metabolism and, hence, fetal nutrition. Certainly, infusing cortisol directly into fetal sheep alters glucose partitioning between uteroplacental and fetal tissues (Ward *et al*. 2004). Moreover, raising corticosterone concentration in pregnant mice to values seen in stressful conditions reduces the glucose transport capacity of the placenta (Vaughan *et al*. 2012, 2015). However, whether maternal cortisol concentrations influence net fetal carbohydrate uptake by regulating uteroplacental carbohydrate metabolism and delivery to the fetus remains unknown.

This study tested the hypothesis that raising maternal cortisol concentrations by exogenous infusion would alter uteroplacental metabolism and glucose transport capacity in pregnant sheep. Specifically, the experiments quantified the uterine uptake, uteroplacental consumption, umbilical uptake and fetal concentrations of glucose and lactate, along with the transplacental clearance of a non‐metabolizable glucose tracer, [^3^H]methyl‐d‐glucose ([^3^H]MeDG). Since glucocorticoids may also influence fetal availability of glucose by altering its rate of endogenous production or insulin‐stimulated utilization, the physiological measurements of nutrient uptake were related to biochemical indices of hepatic glucogenic capacity and tissue insulin signalling in the fetus.

## Methods

### Animals

All procedures were conducted under the Animals (Scientific Procedures) Act 1986. Forty‐three Welsh mountain ewes carrying single fetuses of known gestational age were used in this study. Ewes were housed individually during the experimental period and maintained on concentrate (200 g day^−1^, Beart, Stowbridge, Suffolk, UK) and hay and water *ad libitum*. Food, but not water, was withdrawn for 18–24 h prior to surgery.

### Surgical procedures

Ewes and their fetuses were catheterized under general anaesthesia (2 mg kg^−1^ alfaxalone, i.v. and 2% isofluorane in 5:1 O_2_:NO_2_ inhaled) at day ∼118 of pregnancy (term = 145 days). Catheters were inserted into the uterine vein, and the dorsal aorta and caudal vena cava of both the mother and fetus in all animals and also into the umbilical vein of 24 fetuses, as described previously (Fowden & Silver, 1995). Analgesia (1 mg kg^−1^ carprofen, s.c.) and prophylactic antibiotics (20–30 mg kg^−1^ benzylpenicillin, i.m. mother and i.v. fetus) were administered at the time of surgery. After recovery from anaesthesia, the ewe was returned to her home pen and the catheters were flushed daily until beginning the experimental procedures.

### Experimental procedures

After at least 5 days post‐operative recovery, ewes were infused either with cortisol (1.2 mg kg^−1^ day^−1^ Solucortef, Pharmacia, i.v., *n* = 20) or saline (0.9% w/v, *n* = 21) for a further 5 days, beginning on day ∼125 of pregnancy. Cortisol or saline solutions, which were replaced daily, were infused using an ambulatory infusion pump (MS16A, Graseby, Ashford, UK) contained in a pouch secured to the flank of the ewe. On the final day of infusion (130.1 ± 0.3 days), before the morning feed of concentrates, a metabolic study was conducted to determine steady‐state uterine and umbilical blood flows and substrate uptakes in the subset of animals in which all catheters remained patent (*n* = 24). Antipyrine (80–100 mg ml^−1^ in saline 0.9% w/v) was infused at a known rate (mean 8.3 ± 0.2 mg min^−1^) into the fetal caudal vena cava following an initial priming bolus of ∼5 ml. Simultaneous blood samples were collected from the umbilical vein, fetal aorta, uterine vein and maternal aorta immediately before the start of infusion and ∼120, 140, 160 and 180 min later, when steady state had been reached. Uterine and umbilical blood flows were quantified using the antipyrine steady‐state diffusion technique (Fowden & Hay, 1988; Fowden & Silver, 1995). Net uterine and umbilical uptakes of glucose, lactate and oxygen were then calculated by Fick's principle as the product of blood flow and the arterio‐venous difference in concentration of each substance, determined as below. In 16 animals, [^3^H]methyl‐d‐glucose ([^3^H]MeDG, 40 μCi ml^−1^) was added to the antipyrine infusate in order to measure transplacental glucose clearance on the final day of saline or cortisol infusion (Owens *et al*. 1987).

The simultaneous blood samples were analysed immediately for pH, HCO_3_^−^ concentration and partial pressures of O_2_ and CO_2_, corrected for maternal (37°C) or fetal (39°C) temperature, using an ABL5 Radiometer (Radiometer Copenhagen, Crawley, UK). Blood oxygen content was calculated from the percentage of O_2_ saturation and haemoglobin concentration measured using an ABL80 haemoximeter (also Radiometer Copenhagen). An aliquot (0.5 ml) of each blood sample was then deproteinized with zinc sulphate and barium hydroxide (both 0.15 m) and the remainder transferred to a chilled, EDTA‐coated tube. All samples were then centrifuged (1500 g, 4°C, 5 min) and the supernatants stored at −20°C until required for analysis.

At the end of the experiment, all ewes and fetuses were killed with a lethal dose of anaesthetic (200 mg kg^−1^ sodium pentobarbitone, Pentoject, Animalcare Ltd, Dunnington, York, UK). After checking the correct positioning of all catheters, the fetus and uteroplacental tissues were weighed and placentomes counted and classified into the less everted (A/B) or more everted (C/D) subtype as described previously (Vatnick *et al*. 1991). Samples of placentomes, fetal and maternal liver, and fetal muscle were rapidly frozen in liquid nitrogen then stored at −80°C.

### Biochemical analyses

Whole blood concentrations of antipyrine, glucose and lactate were determined in all five sets of simultaneous samples obtained during the study. Antipyrine concentration was measured in deproteinized blood by the addition of nitrous acid to make 4‐nitroso‐antipyrine, which was determined by spectrophotometry at 340 nm (Edwards, 1959). Glucose was measured colourimetrically in deproteinized blood using glucose oxidase (Fowden & Silver, 1995). Blood lactate content was determined using a YSI 2300 Stat Plus instrument (Yellow Springs, Farnborough, UK). [^3^H]MeDG content was determined in all fetal (0.2 ml) and maternal (0.4 ml) plasma samples by liquid scintillation counting (LKB Wallac Rackbeta). Arterial plasma samples were also analysed for glucose, lactate (YSI 2300 Stat Plus), fructose (Abcam, Cambridge, UK) and α‐amino nitrogen concentrations using previously published methods (Evans *et al*. 1993).

Cortisol and insulin concentrations were determined in maternal and fetal EDTA plasma collected under basal conditions, with the ewe unrestrained in her home pen, on the morning of the study before antipyrine infusion was begun. Cortisol concentration was measured in ethanol‐extracted plasma using a commercially available ELISA (IBL International, Hamburg, Germany) used previously for ovine plasma (Kabaroff *et al*. 2006). Mean recovery of cortisol standard added to stripped sheep plasma was 105 ± 1% while 2‐fold and 4‐fold dilutions of a fetal plasma sample gave corrected values of 94% and 101% of the original concentration, respectively. For two control samples with mean cortisol concentrations of 77 ± 3 and 221 ± 5 ng ml^−1^, the mean inter‐assay and intra‐assay coefficients of variability were 4% and 13%, respectively. The limit of detection of the assay was 2.4 ng ml^−1^. An ovine‐specific ELISA was used for measurement of plasma insulin concentrations (Mercodia, Uppsala, Sweden). The limit of detection was 0.025 ng ml^−1^ and the intra‐assay coefficient of variation was 2%.

Lactate dehydrogenase activity was measured in frozen liver and placentome samples homogenized in 0.25 m sucrose buffer. The homogenate was incubated at 37°C for 10 min in the presence of NADH (0.3 mm). Pyruvate (1.2 m mm) was added to the reaction tube and the rate of oxidation of NADH followed by the change in absorbance at 340 nm for 30 s. Activities of the hepatic gluconeogenic enzymes fructose‐1,6‐bisphosphatase (FBPase) and glucose‐6‐phosphatase (G6Pase), along with hepatic glycogen content were measured as described previously (Fowden *et al*. 1993). Tissue protein content was determined using the Lowry assay (Forhead *et al*. 2003).

### Gene expression

RNA was extracted from maternal and fetal liver and from one placentome of the most abundant subtype from each animal (RNeasy Mini, Qiagen) and reverse transcribed to cDNA (High Capacity Reverse Transcription Kit, Life Technologies). The expression of the glucocorticoid receptor (NR3C1, forward (F)‐CAAGCTGGAATGAACCTGGAA, reverse (R)‐AAGTTTCTTGCGAGACTCCTG) and 11β‐hydroxysteroid dehydrogenase enzymes (HSD1, F‐GATGGGAGCTCACGTGGTAG, R‐CTCCAGGCAGCGGGATAC; and HSD2, F‐CCGGCTGGATCGTGTTGTC, R‐GTTGCCAAAACCAGAGTCACA) was quantified in all three tissues. Additionally, placentome tissue was assessed for expression of the facilitative glucose transporters (SLC2A1, F‐CATGTATGTGGGGGAGGTGT, R‐TGGTTGCCCATGATGGAGT; SLC2A3, F‐CAGCTCTCTGGGATCAACGC, R‐TGACCACACCTGCACCGATA; and SLC2A8, F‐GATGGTGGTCACAGGCATCC, R‐ GGTCTCGGGCATGAAACACA; Limesand *et al*. 2004). Liver expression was quantified for the gluconeogenic enzymes (G6PC, F‐TGTCTGCCTGTCACGAATCT, R‐ TCTGGATGTGGCGGAAAGTC; and PCK1, F‐GGAGGAGGGTGTGATCAAGAG, R‐ CAATTCTGGCCACATCCCTGG; Thorn *et al*. 2009). Real‐time PCR was carried out using MESA green reagents (Eurogentec) and the Light Cycler 480 instrument (Roche). Expression level in unknown samples was determined relative to a standard curve of pooled, 2‐fold serial diluted cDNA and normalized to the geometric mean of glyceraldehyde‐3‐phosphate dehydrogenase (GAPDH, F‐TGGTGAAGGTCGGAGTGAAC, R‐ACGATGTCCACTTTGCCAGT) and hypoxanthine phosphoribosyl transferase (HPRT1, F‐TATGCTGAGGATTTGGAGAAGGT, R‐ATCACATCTCGAGCCAGTCG) abundance. Amplicon size for each primer pair was verified by running the PCR product on an agarose gel.

### Protein abundance

Total protein was extracted from placenta, fetal liver and skeletal muscle (*n* = 7 per group). Protein extracts resolved by electrophoresis were transferred to nitrocellulose membrane and probed with antibodies specific to the insulin receptor (Santa Cruz Biotechnology, Dallas, TX, USA), Akt and phosphorylated Akt (Ser473) (Cell Signaling, Danvers, MA, USA). Protein abundance was quantified from the intensity of specific bands visualized by enhanced chemiluminesence reaction.

### Statistical analyses

Results are presented as means ± SEM. Parametric data for cortisol‐infused and saline‐infused animals were compared by Student's *t* test. The distribution of placentomes between A/B and C/D classifications in the two experimental groups was compared by chi‐squared test. The interdependence of umbilical glucose uptake, maternal and fetal plasma glucose and the transplacental plasma glucose gradient was determined by Pearson correlation. In all cases, significance was taken at the level *P* < 0.05.

## Results

### Maternal and fetal plasma hormone and metabolite concentrations and blood gas status

Maternal cortisol infusion caused a significant increase in plasma cortisol concentration in the mother, but not the fetus, on the fifth day of treatment, compared to saline infusion (Table 1). Plasma concentrations of insulin, glucose and lactate were also raised in cortisol‐ relative to saline‐infused ewes (Table 1). In contrast, these plasma concentrations were unaffected in the fetus by maternal treatment, although maternal cortisol infusion did increase fetal plasma concentrations of fructose compared to controls (Table 1). There was no significant difference between the two groups of ewes in the transplacental concentration gradient for plasma glucose (saline, 2.42 ± 0.10 mmol l^−1^, *n* = 19; cortisol, 2.72 ± 0.12 mmol l^−1^, *n* = 17; *P* > 0.05) or lactate (saline −1.17 ± 0.31 mmol l^−1^, *n* = 19; cortisol, −1.26 ± 0.27 mmol l^−1^ *n* = 17; *P* > 0.05). There was also no difference in maternal and fetal α‐amino nitrogen concentrations with treatment (Table 1). The pH, $P_{CO_{2}}$ and HCO_3_^−^ concentration of maternal blood, but not fetal blood, were higher in cortisol‐ than saline‐infused animals (Table 1). There were no significant differences in $P_{O_{2}}$, haemoglobin content or oxygen saturation of maternal or fetal arterial blood, between saline‐ and cortisol‐infused sheep (Table 1).

**Table 1:** Mean ± SEM maternal and fetal arterial plasma hormone and metabolite concentrations, and arterial blood gas status of saline‐ (*n* = 8–19) and cortisol‐ (*n* = 17–20) infused sheep, on the fifth day of treatment, at day 130 of pregnancy

	Mother		Fetus	
	Saline	Cortisol	Saline	Cortisol
Plasma hormones				
Cortisol (ng ml^−1^)	19.0 ± 5.7	41.1 ± 3.4*	16.3 ± 1.4	24.9 ± 5.0
Insulin (pg ml^−1^)	0.213 ± 0.027	0.541 ± 0.090*	0.133 ± 0.012	0.137 ± 0.01
Plasma metabolites				
Glucose (mmol l^−1^)	3.29 ± 0.11	3.68 ± 0.13*	0.88 ± 0.04	0.97 ± 0.04
Lactate (mmol l^−1^)	0.67 ± 0.07	0.84 ± 0.05*	1.84 ± 0.31	2.10 ± 0.26
α‐Amino nitrogen (mmol l^−1^)	2.43 ± 0.37	2.17 ± 0.26	3.97 ± 0.54	5.10 ± 0.41
Fructose (mmol l^−1^)	^†^	^†^	1.58 ± 0.21	2.68 ± 0.12*
Blood gases				
pH	7.498 ± 0.008	7.525 ± 0.007*	7.352 ± 0.009	7.327 ± 0.013
$P_{O_{2}}$ (mmHg)	96.2 ± 1.4	94.6 ± 2.7	17.9 ± 0.7	18.3 ± 0.6
$P_{CO_{2}}$ (mmHg)	33.7 ± 0.5	37.4 ± 1.1*	52.5 ± 0.8	54.9 ± 0.9
HCO_3_^−^ (mmol l^−1^)	27.2 ± 0.9	30.1 ± 0.7*	27.8 ± 0.9	27.8 ± 0.8
Hb (g dl^−1^)	9.3 ± 0.3	8.7 ± 0.3	9.9 ± 0.4	10.0 ± 0.5
Hb saturation (%)	93.8 ± 0.5	93.8 ± 0.7	51.8 ± 2.5	49.2 ± 1.9
Haematocrit (%)	27.5 ± 0.9	28..2 ± 0.7	31.2 ± 1.3	29.8 ± 1.2

^*^ *P* < 0.05 *versus* saline (*t *test). ^†^Maternal plasma fructose concentrations were not measured as they are typically ≤ 0.1 mmol l^−1^ and below the limit of detection of the assay used (Meznarich *et al*. 1987; Regnault *et al*. 2010).

### Biometry and blood flow

Maternal cortisol infusion had no effect on the crown–rump length, body weight or weight of individual organs of fetuses delivered at day 130 (Table 2). Neither was there a difference in the total weight and number of placentomes, or mean placentome weight between cortisol‐ and saline‐infused animals (Table 2). In addition, there was no difference between saline‐ and cortisol‐infused sheep in mean frequency of A/B (saline 75 ± 8%, *n* = 21; cortisol 71 ± 34%, *n* = 20) or C/D (saline 25 ± 8%; cortisol 29 ± 8%; *P* > 0.05 both cases, *t *test) types of placentomes. Umbilical and uterine blood flows did not differ between saline‐ and cortisol‐infused ewes (Table 3).

**Table 2:** Mean ± SEM fetoplacental biometrical measurements from saline‐ (*n* = 21) and cortisol‐ (*n* = 20) infused sheep on the fifth day of treatment, at day 130 of pregnancy

	Saline	Cortisol
Crown–rump length (cm)	43 ± 1	44 ± 1
Weight		
Fetus (kg)	2.8 ± 0.1	3.0 ± 0.1
Heart (g)	17 ± 1	19 ± 1
Lungs (g)	74 ± 6	87 ± 6
Liver (g)	82 ± 5	100 ± 7
Kidneys (g)	17 ± 1	19 ± 1
Perirenal fat (g)	12 ± 1	11 ± 1
Adrenals (mg)	332 ± 31	313 ± 21
Brain (g)	38 ± 2	41 ± 1
Total placentomes (g)	305 ± 21	300 ± 19
Mean placentome (g)	4.1 ± 0.4	4.3 ± 0.3
Fetus:Placenta	9.9 ± 0.5	10.4 ± 0.5
Total placentome number	96 ± 21	77 ± 3

**Table 3:** Mean ± SEM values of uterine and umbilical blood flows and metabolite fluxes in saline‐ (*n* = 15) and cortisol‐ (*n* = 9) infused sheep infused sheep on the fifth day of treatment, at day 130 of pregnancy

	Saline	Cortisol
Blood flow		
Umbilical (ml min^−1^)	615 ± 33	573 ± 55
Umbilical (ml min^−1^ kg^−1^)	218 ± 14	193 ± 20
Uterine (ml min^−1^)	1275 ± 70	1290 ± 81
Uterine (ml min^−1^ kg^−1^)	400 ± 14	389 ± 30
Blood oxygen content (mmol l^−1^)		
Umbilical vein	4.65 ± 0.16	4.61 ± 0.42
Umbilical artery	3.17 ± 0.18	2.72 ± 0.28
Umbilical vein–artery	1.48 ± 0.08	1.89 ± 0.22
Uterine artery	5.18 ± 0.21	5.16 ± 0.25
Uterine vein	3.85 ± 0.19	3.73 ± 0.24
Uterine artery–vein	1.27 ± 0.13	1.43 ± 0.09
Umbilical oxygen uptake (μmol min^−1^)	892 ± 46	1031 ± 111
Uterine oxygen uptake (μmol min^−1^)	1676 ± 244	1840 ± 154
Uteroplacental oxygen consumption (μmol min^−1^)	784 ± 228	809 ± 182
Blood glucose (mmol l^−1^)		
Umbilical vein	0.97 ± 0.04	0.92 ± 0.07
Umbilical artery	0.82 ± 0.04	0.81 ± 0.07
Umbilical vein–artery	0.15 ± 0.01	0.12 ± 0.02*
Uterine artery	2.44 ± 0.12	2.26 ± 0.04
Uterine vein	2.28 ± 0.12	2.10 ± 0.05
Uterine artery–vein	0.15 ± 0.01	0.16 ± 0.02
Umbilical glucose uptake (μmol min^−1^)	94.2 ± 7.6	65.2 ± 9.9*
Uterine glucose uptake (μmol min^−1^)	190.4 ± 15.1	201.8 ± 20.4
Uteroplacental glucose consumption (μmol min^−1^)	96.2 ± 15.4	136.6 ± 20.3
Blood lactate (mmol l^−1^)		
Umbilical vein	1.29 ± 0.08	1.79 ± 0.32
Umbilical artery	1.21 ± 0.09	1.54 ± 0.23
Umbilical vein–artery	0.08 ± 0.02	0.25 ± 0.11*
Uterine vein	0.52 ± 0.06	0.58 ± 0.05
Uterine artery	0.49 ± 0.06	0.52 ± 0.05
Uterine vein–artery	0.04 ± 0.01	0.06 ± 0.02*
Umbilical lactate uptake (μmol min^−1^)	51.5 ± 11.8	141.2 ± 57.7
Uterine lactate output (μmol min^−1^)	42.8 ± 4.8	74.0 ± 17.4*
Uteroplacental lactate production (μmol min^−1^)	94.3 ± 13.3	215.2 ± 67.7*

^*^ *P* < 0.05 *versus* saline (*t *test).

### Fetoplacental metabolism

#### Oxygen

**Figure 1:**
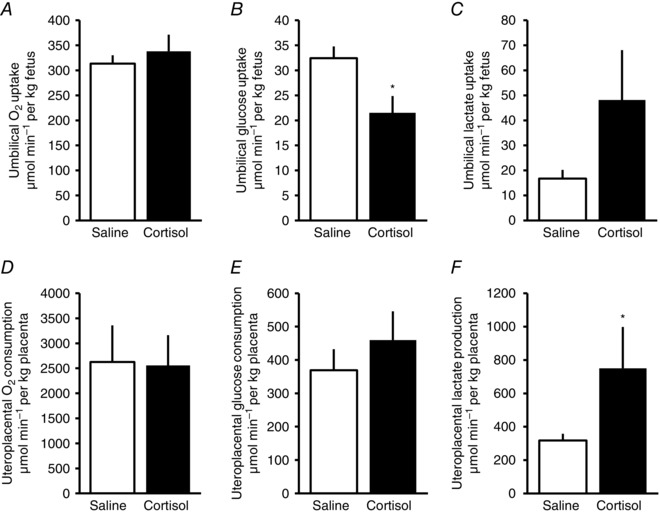
**Mean ± SEM umbilical uptake (*A*–*C*) and uteroplacental consumption/production of oxygen (*A* and *D*), glucose (*B* and *E*) and lactate (*C* and *F*), expressed per kg fetus or per kg placenta, respectively, on the fifth day of infusion with saline (*n* = 15) or cortisol (*n* = 9), at day ∼130 of pregnancy** ^*^ *P* < 0.05 *versus* saline (*t* test).

There was no apparent effect of maternal cortisol infusion on total uterine or umbilical oxygen uptake (Fig. 1 *A* and Table 3). Uteroplacental oxygen consumption also did not differ with maternal treatment either in absolute value or when expressed on a weight specific basis (Table 3 and Fig. 1 *D*).

#### Glucose

Total uterine glucose uptake was similar in the two groups of ewes (Table 4). In contrast, the absolute rate of umbilical glucose uptake was significantly lower in cortisol‐ than saline‐infused animals, due primarily to a significantly smaller umbilical venous–arterial concentration difference in blood glucose (Table 3). Umbilical glucose uptake was also lower in cortisol‐ than saline‐infused sheep when expressed per kg fetus (Fig. 1 *B*). Consequently, it accounted for a significantly smaller percentage of total uterine glucose uptake in cortisol‐treated (34 ± 6%, *n* = 9) than saline‐infused ewes (54 ± 6%, *n* = 15; *P* < 0.05). Conversely, the percentage of total uterine glucose uptake consumed by the uteroplacental tissues was greater in cortisol‐infused ewes (cortisol, 66 ± 6%, *n* = 9; saline, 47 ± 6%, *n* = 15; *P* < 0.05), although absolute and weight specific rates of uteroplacental glucose consumption did not differ significantly with treatment (Table 3 and Fig. 1 *E*).

**Table 4:** Mean ± SEM protein and glycogen contents, and glucogenic enzyme activities in maternal and fetal liver of saline‐ (*n* = 7–10) and cortisol‐ (*n* = 10–20) infused sheep collected at postmortem on the fifth day of infusion, on day 130 of pregnancy

	Maternal liver		Fetal liver	
	Saline	Cortisol	Saline	Cortisol
Protein (mg g^–1^)	159 ± 8	144 ± 3*	124 ± 2	123 ± 3
Glycogen (mg g^–1^)	17 ± 2	39 ± 3*	20 ± 3	21 ± 3
G6Pase activity (U g^–1^)	14.3 ± 1.4	13.1 ± 0.8	1.7 ± 0.3	2.5 ± 0.4
FBPase activity (U g^–1^)	3.4 ± 0.6	5.4 ± 1.0	7.1 ± 0.6	6.7 ± 0.7
LDH activity (U g^–1^)	10.5 ± 1.3	11.0 ± 0.5	8.2 ± 0.7	10.5 ± 0.6*

^*^ *P* < 0.05 *versus* saline (*t *test). FBPase, fructose‐1,6‐bisphosphatase; G6Pase, glucose‐6‐phosphatase; LDH, lactate dehydrogenase.

#### Lactate

Total uteroplacental lactate production was significantly higher in cortisol‐ than saline‐infused ewes (Table 3 and Fig. 1 *F*) due to significant increases in both the umbilical and uterine venous–arterial concentration differences in blood lactate with maternal cortisol treatment (*P* < 0.05, Table 3) Net umbilical lactate uptake varied between fetuses and did not differ significantly between the two groups, either as an absolute value (Table 3, *P* = 0.06) or when expressed per kilogram fetus (Fig. 1 *C*, *P* = 0.07). In contrast, the rate of lactate output into the uterine circulation doubled in cortisol‐infused ewes, compared to the saline‐treated controls (Table 3). However, there was no significant change in the percentage distribution of total uteroplacental lactate production between the umbilical (cortisol, 58 ± 5%, *n* = 9; saline, 51 ± 5%, *n* = 15; *P* > 0.05) and uterine circulations (cortisol, 42 ± 5%, *n* = 9; saline, 49 ± 5%, *n* = 15; *P* > 0.05) with maternal treatment. Placental activity of lactate dehydrogenase was also unaffected by maternal treatment (saline 18.4 ± 1.5 U g^−1^ *n* = 7; cortisol 15.9 ± 1.4 U g^−1^ *n* = 10; *P* > 0.05).

### Placental glucose transport capacity

**Figure 2:**
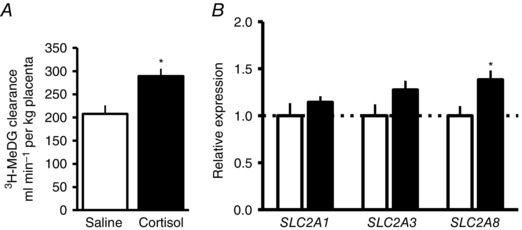
**Mean ± SEM transplacental clearance of [^3^H]methyl‐d‐glucose per kg placenta (*A*, saline *n* = 8, cortisol *n* = 8) and placental glucose transporter gene expression (*B*, saline *n* = 9, cortisol *n* = 15) on day ∼130 of pregnancy** ^*^ *P* < 0.05 *versus* saline (*t* test).

Per kilogram of placenta, transplacental clearance of the non‐metabolizable glucose tracer, [^3^H]MeDG, was 40% greater in cortisol‐ than saline‐treated ewes (Fig. 2 *A*). Concomitantly, placental expression of the facilitative glucose transporter gene, *SLC2A8*, was 38% higher in cortisol‐ than saline‐infused animals (Fig. 2 *B*). In contrast, placental expression of the *SLC2A1* and *SLC2A3* genes was unaffected by maternal treatment (Fig. 2 *B*). There was no significant effect of maternal treatment on placental protein content (saline, 108 ± 9 mg g^−1^, *n* = 7; cortisol, 109 ± 7 mg g^−1^, *n* = 10; *P* > 0.05).

### Hepatic glucogenic capacity

Elevating cortisol concentrations reduced the protein content but increased glycogen content of maternal liver relative to saline infusion on day 130 of pregnancy (Table 4). However, there was no difference in fetal hepatic protein or glycogen contents between the saline and cortisol groups. Neither was there a significant alteration in activity of the gluconeogenic enzymes, glucose‐6‐phosphatase and fructose‐1,6‐bisphosphatase, in either maternal or fetal liver. Cortisol infusion led to significantly higher lactate dehydrogenase activity in fetal, but not maternal, liver than seen with saline infusion (Table 4). There were no significant differences in hepatic *G6PC* or *PCK1* gene expression between treatment groups in either the mother or fetus (*P* > 0.05, data not shown).

### Glucocorticoid bioavailability

Glucocorticoid receptor (*GR*) gene expression did not differ between the saline‐ and cortisol‐infused groups in the placenta, maternal or fetal liver or in fetal skeletal muscle (Table 5). Neither was there an effect of maternal cortisol infusion on gene expression of the glucocorticoid metabolizing enzymes, *HSD1* (placenta, maternal and fetal liver and fetal skeletal muscle) and *HSD2* (placenta only) (Table 5).

**Table 5:** Mean ± SEM relative expression of genes related to glucocorticoid bioavailability in maternal and fetal liver of saline‐ (*n* = 8–10) and cortisol‐ (*n* = 14–20) infused sheep collected at postmortem on the fifth day of treatment, on day 130 of pregnancy

	Saline	Cortisol
Maternal liver		
*GR*	1.00 ± 0.07	0.91 ± 0.03
*HSD1*	1.00 ± 0.04	0.87 ± 0.05
Fetal liver		
*GR*	1.00 ± 0.08	1.10 ± 0.07
*HSD1*	1.00 ± 0.10	1.13 ± 0.10
Fetal muscle		
*GR*	1.00 ± 0.09	1.11 ± 0.18
*HSD1*	1.00 ± 0.09	1.34 ± 0.24
Placenta		
*GR*	1.00 ± 0.07	1.19 ± 0.10
*HSD1*	1.00 ± 0.26	0.84 ± 0.12
*HSD2*	1.00 ± 0.12	0.84 ± 0.11

### Fetal insulin receptor signalling

**Figure 3:**
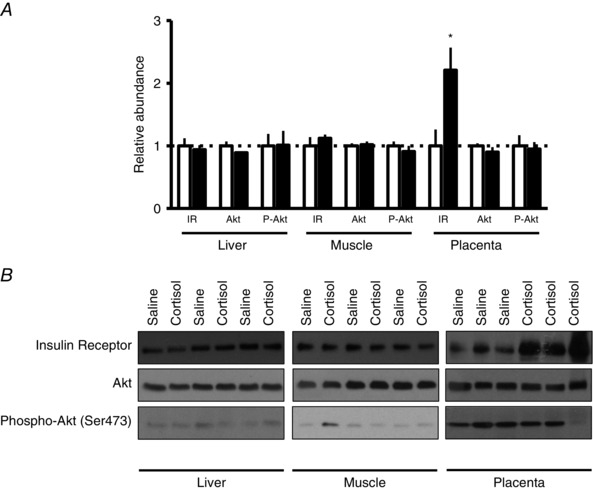
**Mean ± SEM relative abundance (*A*) and representative Western blots (*B*) of insulin signalling proteins in fetal liver, muscle and placenta of saline‐ and cortisol‐infused sheep** ^*^ *P* < 0.05 *versus* saline (*t* test).

Insulin receptor (IR) protein abundance was significantly greater in the placenta of cortisol‐infused animals but did not differ in fetal liver or skeletal muscle, relative to controls (Fig. 3). Maternal cortisol infusion had no effect on fetal hepatic or skeletal muscle Akt protein abundance, or its phosphorylation at the serine‐473 residue, key signalling molecules downstream of the IR (Fig. 3).

## Discussion

This study is the first to show that increasing maternal cortisol concentrations within the physiological range during sheep pregnancy alter uteroplacental metabolism with consequences for the supply of nutrients to the fetus. When maternal plasma cortisol was raised by intravenous infusion, a greater percentage of the total amount of glucose taken up by the gravid uterus was used by the uteroplacental tissues with the result that proportionately less glucose was available for onward transfer to the fetus. The reduction in net umbilical glucose uptake occurred despite increases in placental glucose transporter expression and glucose transport capacity measured as [^3^H]MeDG clearance. Concomitantly, lactate was produced at a higher rate by the uteroplacental tissues of cortisol‐treated ewes and distributed into both the fetal and maternal circulations without any change in uteroplacental consumption or umbilical uptake of oxygen. The cortisol‐induced rise in uteroplacental lactate production was associated with increased maternal but not fetal lactate concentrations and with enhanced lactate dehydrogenase activity in fetal but not maternal liver. Maternal cortisol concentrations, therefore, appear to regulate uteroplacental metabolism and glycolysis, in particular, to vary the availability of different carbohydrates for fetal use.

Cortisol administration increased plasma cortisol concentration in the ewe by 100% on the final day of infusion, in line with observations made during cortisol infusion at the same dose over a more prolonged period in late pregnancy (Jensen *et al*. 2005; Reini *et al*. 2008; Keller‐Wood *et al*. 2014). However, in contrast to these previous studies, fetal cortisol concentrations were not significantly raised after 5 days of maternal cortisol infusion. In the current study, the rise in maternal plasma cortisol concentrations was accompanied by a decline in hepatic protein content and increases in the plasma insulin and glucose concentrations, blood pH and HCO_3_^−^ concentration and in the hepatic glycogen content of the mother, consistent with the known actions of glucocorticoids on adult insulin sensitivity and renal and hepatic metabolism (Holness & Sugden, 2001; Jensen *et al*. 2002 *a*; Franko *et al*. 2007; Koeppen, 2009; Keller‐Wood *et al*. 2014). Since cortisol treatment had no effect on gene expression of GR or 11β‐hydroxysteroid dehydrogenase enzyme isoforms in the placenta, maternal or fetal liver, the maternal and fetal metabolic changes seen with this treatment are most likely to be due to the increased cortisol concentration in the maternal circulation mediated in the fetus by the concomitant changes in uteroplacental metabolism and nutrient delivery.

Net umbilical glucose uptake was reduced by 30% in response to maternal cortisol treatment in the present study, despite increases in both placental GLUT8 expression and [^3^H]MeDG clearance. There were also no changes in the actual rates of uterine glucose uptake, uteroplacental glucose consumption and umbilical blood flow or in the transplacental plasma glucose concentration gradient which could explain the fall in umbilical glucose uptake with treatment. Since uteroplacental lactate production increased more than 2‐fold with maternal cortisol treatment, the most plausible explanation for the decreased umbilical glucose uptake is that glucose is diverted from transplacental transfer into uteroplacental lactate synthesis.

Lactate production can occur either by increased flux through the glycolytic pathway or by diverting pyruvate from oxidative metabolism directly or as a result of impaired mitochondrial function. There was no change in uteroplacental oxygen consumption in the cortisol‐treated ewes in the current study, which suggests that mitochondrial oxidative function was not affected significantly by maternal treatment. Neither was there any change in placental activity of lactate dehydrogenase, the enzyme responsible for interconverting pyruvate and lactate. Since the fall in umbilical glucose uptake with maternal cortisol treatment could account for only about half of the increase in uteroplacental lactate production, either a greater proportion of the glucose normally consumed by the uteroplacental tissues was used for lactate production or lactate was produced from glucose or fructose derived from the fetal circulation. Fetal fructose concentrations were higher in the cortisol‐treated ewes in the present study and previous studies have shown that lactate can be synthesized by uteroplacental tissues from both fetal glucose and fructose in normal, unstressed ewes, albeit at low rates (Sparks *et al*. 1982; Meznarich *et al*. 1987; Hay *et al*. 1990). In normal conditions, fructose is produced endogenously and accumulated at high concentrations within the conceptus, both as an energy source and organic osmolyte (Teng *et al*. 2002; Jauniaux *et al*. 2005). Although it is unclear whether increased fetal fructose concentrations in this study are derived from the placenta or fetal liver (Britton *et al*. 1967; Regnault *et al*. 2010), enhanced fructogenesis is likely to parallel increased blood glucose levels in the ewe and may act to regulate cellular redox potential in the fetus during maternal cortisol infusion (Huggett *et al*. 1951; Jauniaux *et al*. 2005; Brown *et al*. 2014). Since GLUT8 transports glucose and fructose (Debosch *et al*. 2014), upregulation of placental *SLC2A8* expression by maternal cortisol infusion may enhance the availability of both fetal glucose and fructose for uteroplacental lactate synthesis.

Previous studies have also shown increased placental expression of fatty acid and amino acid transporters when maternal glucocorticoids are raised by undernutrition or exogenous administration in sheep and mice, respectively (Fowden & Silver, 1985; Ma *et al*. 2011). This may provide more fat and amino acids as alternative substrates to maintain oxidative metabolism of the uteroplacental tissues in the face of their increased glycolytic use of glucose.

The molecular mechanisms by which cortisol alters uteroplacental metabolism remain largely unknown. In other tissues, aerobic glycolysis is activated by the PI3K–Akt pathway through which insulin and other hormones act (Vander Heiden *et al*. 2009). Even though there were no changes in placental expression or phosphorylation of Akt *per se* in the current study, the enhanced placental IR expression together with the raised maternal insulin concentrations in the cortisol‐treated ewes suggests that activation of the PI3K–Akt signalling pathway may be involved in regulating uteroplacental metabolism. Certainly, in previous studies, altered placental PI3K–Akt signalling accompanies the adaptive changes in placental morphology and nutrient transport activity induced by dietary or other stressful challenges during pregnancy in sheep and mice (Arroyo *et al*. 2010; Zhu *et al*. 2010; Ma *et al*. 2011; Sferruzzi‐Perri *et al*. 2011; Vaughan *et al*. 2015; Higgins *et al*. 2016).

Whatever the mechanisms involved, the cortisol‐initiated switch from supplying carbohydrate to the fetus primarily as glucose to a greater dependence on lactate will have consequences for fetal metabolism and acid–base balance. Lactate can be used for both oxidative and non‐oxidative metabolism in fetal sheep (Sparks *et al*. 1982; Hay *et al*. 1983). It can also be converted to glucose by hepatic gluconeogenesis to supply fetal tissues like the brain, with little capacity to use lactate, particularly when glucose availability is limited (Jones *et al*. 1975; Levitsky *et al*. 1988; Bissonnette *et al*. 1991). The increased lactate dehydrogenase activity in the fetal liver of cortisol‐treated ewes is consistent with the possibility of increased gluconeogenesis from lactate, although there were no changes in expression or activity of key gluconeogenic enzymes in the fetal liver with maternal treatment. The greater fetal provision of lactate with maternal cortisol treatment may be a mechanism to limit the rise in fetal glucose levels in response to the concomitant maternal hyperglycaemia. Indeed, a significant increase in both maternal and fetal lactate concentrations occurs when maternal blood glucose levels are raised over a more prolonged, 10 day period of cortisol infusion in late pregnancy (Jensen *et al*. 2005). In normal conditions, increases in fetal glucose concentrations stimulate fetal insulin secretion and lead to increased fetal glucose uptake and growth, which would be an inappropriate response at a time of maternal stress when cortisol levels are high and more nutrients may be required by maternal tissues (Fowden & Forhead, 2009). Certainly, there were no changes in fetal insulin concentrations or gene expression of IR and Akt in fetal liver or skeletal muscle with maternal cortisol treatment in the current study, which might have altered fetal glucose utilization independently of the glucose supply. Neither did mean fetal body weight differ from control values in this or previous studies of longer cortisol infusions, although growth of individual fetuses, measured as girth increment, is reduced by the end of 10 days of maternal cortisol treatment (Jensen *et al*. 2002 *b*; Reini *et al*. 2008; Keller‐Wood *et al*. 2014).

In summary, maternal cortisol treatment adapts uteroplacental metabolism by increasing both glycolytic production of lactate and oxidative use of substrates in addition to glucose. Since the total net umbilical supply of carbohydrate carbon was unaffected by maternal treatment, the cortisol‐induced switch in uteroplacental metabolism to provide the fetus with less glucose and more lactate carbon may allow more maternal control over fetal metabolism and growth while still providing the fetus with an adequate carbohydrate supply to meet its immediate needs. Indeed, in increasing lactate production aerobically, the uteroplacental tissues of the cortisol‐treated ewes may be reverting to the metabolic strategy of the early embryo in using aerobic glycolysis to accumulate biomass and regulate the redox state of metabolically active, proliferating cells (Jauniaux *et al*. 2005; Krisher & Prather, 2012). Overall, the findings indicate that the ovine placenta can integrate maternal signals of adversity with fetal signals of nutrient demand in determining its metabolic phenotype. Whilst these adaptations in uteroplacental metabolism may be beneficial for fetal and maternal survival in the short term, prolonged uteroplacental production and fetal delivery of lactate, for instance, may have adverse consequences in the longer term and explain previous findings of poor fetal viability in ewes infused with cortisol for several weeks during late pregnancy (Keller‐Wood *et al*. 2014).

## Additional information

### Competing interests

The authors have no competing interests.

### Author contributions

O.R.V. and A.L.F. conceived of and designed the experiments, drafted the article and revised it critically for important intellectual content. All authors collected, analysed and interpreted the data. All authors have approved the final version of the manuscript and agree to be accountable for all aspects of the work. All persons designated as authors qualify for authorship, and all those who qualify for authorship are listed.

### Funding

The studies described in this manuscript were supported by the Biotechnology and Biological Sciences Research Council, grant number BB/I011773/1.
